# The association between HIV-1 Tat and Vif amino acid sequence variation, inflammation and Trp-Kyn metabolism: an exploratory investigation

**DOI:** 10.1186/s12879-024-09874-0

**Published:** 2024-09-09

**Authors:** Monray E. Williams, Levanco K. Asia, Zander Lindeque, Esmé Jansen van Vuren

**Affiliations:** 1 Human Metabolomics, North-West University, Potchefstroom, South Africa; 2 Hypertension in Africa Research Team (HART), North-West University, Potchefstroom, South Africa; 3 South African Medical Research Council Unit for Hypertension and Cardiovascular Disease, North-West University, Potchefstroom, South Africa

**Keywords:** Viral proteins, Inflammation, Pathogenesis, Tryptophan-Kynurenine Pathway, Metabolomics

## Abstract

**Background:**

HIV-1 has well-established mechanisms to disrupt essential pathways in people with HIV, such as inflammation and metabolism. Moreover, diversity of the amino acid sequences in fundamental HIV-1 proteins including Tat and Vif, have been linked to dysregulating these pathways, and subsequently influencing clinical outcomes in people with HIV. However, the relationship between Tat and Vif amino acid sequence variation and specific immune markers and metabolites of the tryptophan-kynurenine (Trp-Kyn) pathway remains unclear. Therefore, this study aimed to investigate the relationship between Tat/Vif amino acid sequence diversity and Trp-Kyn metabolites (quinolinic acid (QUIN), Trp, kynurenic acid (KA), Kyn and Trp/Kyn ratio), as well as specific immune markers (sCD163, suPAR, IL-6, NGAL and hsCRP) in *n* = 67 South African cART-naïve people with HIV.

**Methods:**

Sanger sequencing was used to determine blood-derived Tat/Vif amino acid sequence diversity. To measure Trp-Kyn metabolites, a LC–MS/MS metabolomics platform was employed using a targeted approach. To measure immune markers, Enzyme-linked immunosorbent assays and the Particle-enhanced turbidimetric assay was used.

**Results:**

After adjusting for covariates, sCD163 (*p* = 0.042) and KA (*p* = 0.031) were higher in participants with Tat signatures N24 and R57, respectively, and amino acid variation at position 24 (adj R^2^ = 0.048, β = -0.416, *p* = 0.042) and 57 (adj R^2^ = 0.166, β = 0.535, *p* = 0.031) of Tat were associated with sCD163 and KA, respectively.

**Conclusions:**

These preliminary findings suggest that amino acid variation in Tat may have an influence on underlying pathogenic HIV-1 mechanisms and therefore, this line of work merits further investigation.

**Supplementary Information:**

The online version contains supplementary material available at 10.1186/s12879-024-09874-0.

## Introduction

HIV-1 remains a matter of global significance and concern, even after over 40 years of research. As of 2022, nearly 38 million individuals were infected with HIV-1 [1]. The virus is categorized into groups M, N, O, and P according to genetic differences [2]. Among these groups, group M is the most prevalent worldwide and is further divided into subtypes A, B, C, D, F, G, H, J, and K [3]. The pathophysiology of HIV-1 varies by region and subtype. Among several contributing mechanisms, subtype-specific alterations of viral protein amino acid sequences are considered influential in HIV-1 pathophysiology and clinical outcomes in people with HIV [4, 5]. HIV encodes 15 unique proteins, which can be broadly categorized into structural, enzymatic, regulatory, and accessory. Structural proteins include the Gag polyproteins: p17 (Matrix), p24 (Capsid), p7 (Nucleocapsid), and p6, and the envelope polyproteins, which include gp120 (Surface glycoprotein) and gp41 (Transmembrane glycoprotein). Enzymatic proteins include the Pol polyproteins: Protease, Integrase, Reverse Transcriptase, and RNase H. Regulatory proteins include Tat (Transactivator of Transcription) and Rev (Regulator of Expression of Virion Proteins), while accessory proteins include Vif (Viral Infectivity Factor), Nef (Negative Regulatory Factor), Vpu (Viral Protein U), and Vpr (Viral Protein R) [6, 7]. Specific amino acid variations in these proteins have been associated with differing pathogenesis [8–11].

Among the myriad of HIV-1 viral proteins, Tat [12, 13] and Vif [14, 15] have garnered significant attention for their role in HIV-1 pathogenesis. The HIV-1 Tat protein binds to RNA element called the Trans-activation response element (TAR) and enlists CDK9/cyclin T1 along with other host factors to trigger HIV-1 transcription, playing a pivotal role in HIV pathogenesis. [16]. Likewise, Vif fulfils functional roles by counteracting members of the Apolipoprotein B Editing Complex (APOBEC)3 (A3) family, directing them for degradation. The A3 proteins act as host antiviral cellular proteins, inducing hypermutations in the viral genome [17].

These viral proteins can still be detected despite effective antiretroviral therapy and undetectable viremia, suggesting they may have a functional role in the modern combined antiretroviral therapy (cART) era and warrant further investigation [18, 19]. Specific amino acids in Tat and Vif are linked to negative clinical outcomes in people with HIV, such as disease progression [19, 20]. Variation of Tat at position 24 may influence proviral DNA load and CD4^+^ counts [21]. The amino acid sequence variation of the Tat protein at position 31 is one of the most widely investigated positions, as the disulfide motif (C30C31) has been implicated in higher levels of inflammation in cell culture studies [22–24]. The position 57 of Tat has also been implicated in the dysregulation of transactivation [25, 26] and inflammation [20, 27]. For Vif, variation at position 17 between Lysine and Arginine was suggested to have a role in viral infectivity and may function in binding APOBEC3G [28]. Lastly, Vif mutations at position 31 between Valine and Isoleucine are involved in cell cycle arrest by inducing the degradation of protein phosphatase 2 regulatory subunit B family (PPP2R5) subunits [29, 30]. Thus, previous studies highlight that these specific amino acid signatures may be important in the pathogenesis of HIV-1.

In line with this, previous investigations by our group, conducted in a limited cohort of South African individuals living with HIV, have shown that Tat amino acid sequence diversity influences the levels of peripheral chemokine ligand 2 (CCL2) and thymidine phosphorylase (TYMP) [20]. Additionally, specific amino acids in the Vpr have been shown to influence the levels of certain immune markers and metabolites in the tryptophan-kynurenine (Trp-Kyn) pathway [31, 32]. Therefore, we hypothesized that other viral proteins might also influence specific immune markers and metabolites. Building on these initial findings and conducting an exploratory analysis, we aimed to determine whether Tat sequence variations could affect other immune markers/metabolites not previously investigated. Additionally, we recently demonstrated that Vif in South Africa is genetically diverse compared to other regions where subtype C is present [33]; however, the clinical implications of these amino acid variants remain unknown. Thus, we sought to explore whether Vif amino acid sequence variants could influence immune markers and metabolites within this study. Further, our understanding of these proteins and the effects of amino acid sequence variations come from studies in regions dominated by subtype B (Europe and United States of America)[34]. This is intriguing since subtype C (Southern African region) has the highest global incidence, yet sequence variation knowledge in such cohorts is notably sparse [20, 21, 33, 35].

Moreover, dysregulated inflammation [36] and metabolism are crucial mechanisms that contribute to adverse clinical outcomes in people with HIV [37, 38]. Our focus was specifically on the Trp-Kyn metabolic pathway, particularly examining kynurenic acid (KA), quinolinic acid (QUIN), Trp, and Kyn, as these metabolites has been previously linked to HIV-1 pathogenesis [39, 40]. Further, current evidence has suggested a correlation between peripheral inflammation and the Trp-Kyn metabolites [41] in people with HIV [32]. In general, our understanding of the influence of viral protein amino acid sequence diversity on underlying mechanisms is largely based on studies conducted in cohorts where subtype B predominates [34]. Therefore, there is a need to profile the influence of viral protein amino acid sequence diversity in subtype C-specific cohorts. In this study, we aimed to use an exploratory approach to investigate the potential associations between specific Tat and Vif amino acid sequence variants with soluble urokinase plasminogen activator receptor (suPAR), interleukin (IL)-6, high-sensitivity C-reactive protein (hsCRP), soluble CD163 (sCD163) and neutrophil gelatinase-associated lipocalin (NGAL), as well as Trp-Kyn pathway metabolites in a cART treatment-naive South African cohort.

## Methods

### Study cohort

**Fig. 1:**
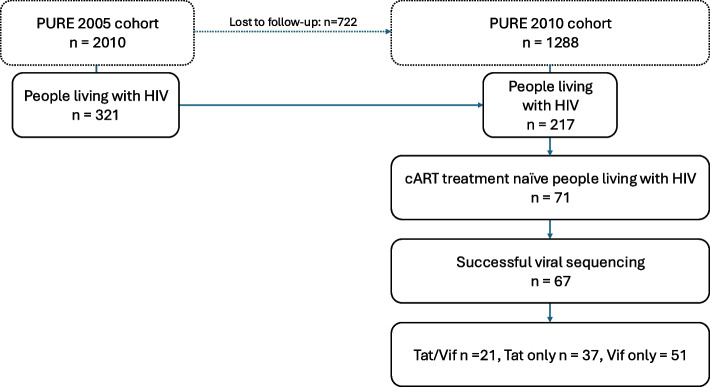
Study layout

The Prospective Urban and Rural Epidemiology (PURE) study is centred on cardiovascular diseases (CVD) and investigates the underlying mechanisms that may influence CVD development, including factors like HIV and inflammation. This comprehensive study spanned 20 countries worldwide, encompassing nations with diverse income levels, ranging from high to low-income categories [42]. As a component of this comprehensive investigation, individuals aged 30 years and above, of African descent, were recruited from both rural and urban areas in the North West province of South Africa. Participants were assisted by trained fieldworkers in an interview-manner to obtain questionnaire data in the home language of the participant. A questionnaire tailored and standardized according to the international PURE study and adapted to each country was utilized to gather demographic and lifestyle information from participants [42]. Demographic data covered age and gender, while self-reported lifestyle details encompassed location, employment, medication usage, alcohol consumption, and tobacco use. Data at baseline was voluntarily collected in 2005, involving *n* = 2010 participants. Data was then collected in 2010 (1288 participants) serving as the first follow-up. This study included participants who were HIV-1 positive and not on treatment (cART-naïve) at the time of data collection in 2010, comprising a total of 71 participants. For the subsequent analysis, we included only those participants for whom viral sequencing was successful. Thus, 67 people with HIV were included for further analysis (Fig. 1). We were particularly interested in specific amino acid variations within the Tat and Vif proteins due to their pathogenic roles in HIV-1. Therefore, for Tat, we investigated amino acid variations at position 24 (Lysine (K) vs. Asparagine (N)), position 31 (Cysteine (C) vs. Serine(S)), position 57 (Arginine (R) vs. Serine(S)), and position 68 (Leucine (L) vs. Proline (P)). This study protocol was approved by the Health Research Ethics Committee of North-West University in South Africa (NWU-00106–22-A1).

### HIV Status

Before conducting HIV testing, all participants received counselling from a trained counsellor. The initial step in determining their HIV status involved using the First Response rapid HIV card test (Premier Medical Corporation Limited, Daman, India), following the protocol outlined by the South African Department of Health. A confirmatory test, the SD BIOLINE HIV 1/2 3.0 card test (Standard Diagnostics, INC in Korea), was then conducted. If participants tested positive for HIV, they then received post-counselling and were referred to the nearest clinical/hospital for further assessment. At the clinic/hospital, CD4 + counts were analysed using the flow cytometric method (Beckman COULTER EPICS XL™ machine, Fullerton, USA).

### Preparation of blood samples

After fasting, whole blood samples were collected in EDTA tubes. To isolate plasma, samples underwent centrifugation at 2000 × g for 15 min at 10 °C within a 2-h timeframe. After centrifugation, the samples were transferred into microcentrifuge tubes, promptly frozen using dry ice, and subsequently stored at -80 °C until further analysis. For samples gathered from rural locations, the same rapid freezing process was employed, but they were maintained at -18 °C for a maximum of five days before transportation to the laboratory. Upon arrival, these samples were again stored at -80 °C until they underwent subsequent analysis.

### Targeted metabolomics analysis

All chemicals and standards used in the analyses were described previously [32]. We examined Trp-Kyn metabolic profiles, focusing specifically on Trp, QUIN, Kyn, Trp/Kyn ratio, and KA, due to their potential role in HIV-1 pathophysiology. These were investigated using high-performance liquid chromatography (HPLC) and quantified through liquid chromatography–tandem mass spectrometry (LC–MS) using a targeted approach. To precipitate proteins from the HIV plasma samples, 300 µl of ice-cold acetonitrile was added to 100 µl of the plasma, along with 100 µl of an internal standard mixture (10 ppm) containing L-Kynurenine-d4 [2-aminophenyl-3,5-d2], Kynurenic acid-d5, D-Tryptophan (Indole-D5, 98%) and 2,3-pyridinedicarboxylic acid-d3 (Major) [53]. Matrix-appropriate external calibrators were prepared in a similar fashion. These calibrators were enriched with Kyn, Trp and QUIN. To establish a calibration curve, these calibrators were diluted serially. Thereafter, all samples underwent vortexing and placed on ice for 10 min to allow protein precipitation, followed by centrifugation at 12,000 × g for 10 min. The supernatant obtained after centrifugation was collected, dried (nitrogen gas), and stored at -80 °C until analysis. Before analysis, the samples were thawed to room temperature. Thereafter samples were re-dissolved in 100 µl of mobile phase (50% HPLC water and 50% acetonitrile), incubated for 30 min at room temperature, and underwent one additional vortexing step. Samples were then transferred to glass vials with vial inserts for LC–MS/MS analysis. We assessed the metabolic profiles utilizing multiple reaction monitoring (MRM) with electrospray ionization mass spectrometry in both positive and negative ionization modes. With the use of commercial standards, we further optimized the MRM transitions and chromatographic conditions on the LC–MS/MS. This was to ensure precise detection and quantification of the target metabolites. The Kyn to Trp ratio was utilized to estimate IDO activity [43].

### LC–MS/MS analyses

HIV-positive plasma samples underwent targeted LC–MS/MS analysis using an Agilent Technologies 6470 triple quadrupole mass spectrometer coupled with a 1200 series HPLC system. Chromatographic separations were performed on an Acquity UPLC CSH C18 1.7µm 2.1 × 100mm column maintained at 80°C, with a 1 µL sample injection. The mobile phases, HPLC water (solvent A) and acetonitrile (solvent B), both contained 0.1% formic acid. The positive and negative polarization characteristics were previously described [32].

### Immune marker measurement in blood samples

We prepared samples following the procedure outlined in Sect. "Preparation of blood samples". We focused on examining specific immune markers, namely NGAL, suPAR, hsCRP, IL-6 and sCD163. We selected these markers due to their potential significance in inflammation-related diseases in people with HIV. More specifically, the levels of these markers are known to be influenced by HIV-1 [44–49]. Previous work done by our group has shown that, in a South African cohort, certain Tat amino acids (R57S) were linked to participants having lower peripheral CCL2 and TYMP levels [20]. To build on these initial findings, it is reasonable to hypothesize that Tat and Vif amino acid variations may influence other immune markers. To quantify plasma suPAR levels, the suPARnostiC® ELISA kit (ViroGates, Copenhagen, Denmark) was used. Plasma hsCRP were analysed using a particle-enhanced turbidimetric assay (Cobas Integra 400 plus, Roche Diagnostic, Basel, Switzerland), while IL-6 levels were determined via the electrochemiluminescence immunoassay method (Elecsys 2010, Roche, Basel, Switzerland). ELISA assays (R&D Systems DuoSet) were employed for plasma sCD163 and NGAL measurements, following the instructions from the manufacturer, with all samples analysed in duplicate. The coefficients of variation for both intra- and inter-assay tests fell within acceptable ranges, with values below 8% and 10%, respectively.

### Viral sequencing

Viral sequencing was conducted as previously described [31, 32]. In brief, RNA was extracted from plasma, reverse transcribed via polymerase chain reaction (PCR), and the cDNA was used to amplify the specific Tat/Vif regions (HXB2 position 4900–6351). The following primers were utilized to amplify this region: Vif-1 (5’GGGTTTATTACAGGGACAGCAGAG) and CATH-4R (5’-GTACCCCATAATAGACTGTGACC). The PCR cycling conditions have been detailed in our primary investigations [31, 32]. After PCR amplification, the PCR products were purified and sequenced using the BigDye Terminator and analysed with the ABI Prism 3130xl automated DNA sequencer (Applied Biosystems, Foster City, CA). The acquired sequences underwent analysis utilizing GeneStudio™ Professional sequence analysis software (Version 2.2) and were subsequently translated into amino acid sequences employing the Expasy translate tool [50]. Key mutations within the Tat/Vif regions were identified and specifically highlighted for further analysis.

### Statistical approach

All analyses were carried out utilizing SPSS software (IBM, USA), version 29. *P*-values below 0.05 were deemed statistically significant for all analyses. Normality of variables was evaluated by visually inspecting QQ plots alongside descriptive statistics. It was observed that the data distribution of immune markers IL-6, hsCRP, and sCD163, as well as the metabolite KA, exhibited skewness. Consequently, the data for skewed variables were log-transformed prior to statistical analyses. After log transformation, all test assumptions were checked and met. Data presented acceptable skewness and kurtosis values within the range of -2 and 2, residual plots indicated homoscedasticity and linearity. The Durbin-Watson statistic was within acceptable range, indicating independence of errors. Lastly, residuals of the regression models were normally distributed.

Primarily, we aimed to evaluate whether specific immune marker/metabolite levels had a relationship with single amino acid variants in Tat and Vif, respectively. Therefore, we divided participants into groups for Tat: K24 vs. N24, C31 vs. S31, R57 vs. S57, and L68 vs. P68. We divided participants into groups for Vif: K17 vs. R17 and I31 vs. V31. These amino acid positions were selected as previous studies have implicated it in pathogenesis and neuropathogenesis of HIV-1 [9, 21, 27, 28, 33]. χ2 tests were utilized to assess group disparities across amino acid variants for sex, gender, smoking, alcohol consumption, and locality. Independent sample T-tests were utilized to detect differences in study characteristics (such as age, body mass index (BMI), and CD4 + counts), as well as levels of immune markers/metabolites. For the χ2 tests and independent sample t-tests, *p* values of < 0.05 we deemed significant. To correct for the number of immune markers or metabolites tested, a Bonferroni correction was implemented (α/n = 0.05/5 = 0.01) in all relevant analyses. Pearson correlation analysis was utilized to identify covariates by exploring correlations between sociodemographic and lifestyle variables (age, sex, BMI, smoking status, alcohol use, and locality) and specific immune markers/metabolites. An Analysis of Covariance (ANCOVA) was used to adjust for the influence of covariates, which helps in isolating the effect of the independent variable on the dependent variable. Therefore, an ANCOVA was performed, with immune marker/metabolite levels as the dependent variables, to compare their levels among Tat or Vif amino acid variants. Adjustments were made for sex, BMI, and locality in the investigation of immune markers, and for alcohol use and BMI in the examination of metabolites to prevent model overfitting. Multiple regression analysis using the enter method was employed to determine associations between Tat/Vif amino acid variants and immune marker/metabolite levels after adjusting for covariates. For the Pearson correlation and multiple regression analyses, p values of < 0.05 we deemed significant.

## Results

### Study characteristics

This study included a total of *n* = 67 treatment naïve people with HIV, with a mean age of 47.23 years as shown in Table 1. A total of 70% (*n* = 47) of the cohort were men. Viral load data was not documented in the primary study; nonetheless, at the time of sample collection, all participants were treatment naive. Only 59% of participants had CD4 + count data, with a mean CD4 + count of 299.5 (148.7) cells/μl. Approximately half of the participants (*n* = 34, 51%) were recruited from urban areas in South Africa. Self-reported data on alcohol consumption and smoking status were accessible for 99% (*n* = 66) of the participants. Among these individuals, 62% and 41% were identified as current or former smokers and alcohol consumers, respectively. Participants with available data for both Tat/Vif sequences totalled *n* = 21, while those with Tat only were *n* = 37, and Vif only were *n* = 51. Immune markers were examined in the entire cohort (*n* = 67); however, due to limited availability of biological samples, metabolomic analysis was conducted on a subset of the entire cohort (*n* = 48).

**Table 1:** Study characteristic of people with HIV

**Characterising variable**	**Value / measurement for overall cohort** (*n* = 67)	**Value / measurement for cohort with metabolite data** (*n* = 48)
Age in years, mean (SD)	47.23 (7.0)	47.44 (6.5)
Sex, female N (%)	47 (71%)	32 (66.7)
CD4^+^, mean (SD)	299.5 (148.7)	295.7 (177.4)
Locality, rural N (%)	33 (47.1)	22 (45.8)
Smoking, N (%)	41 (62.1)	29 (47)
Alcohol consumption, N (%)	28 (42.4)	22 (45.8)
Body mass index, mean (SD)	23.26 (5.7)	23.1 (5.2)
IL-6, pg/mL, mean (SD)	7.9 (9.2)	8.8 (10.5)
hsCRP, mg/L, mean (SD)	10.6 (25.5)	12.7 (29.3)
suPAR, ng/mL, mean (SD)	4.2 (1.6)	4.3 (1.7)
NGAL, μg/ml, mean (SD)	52.4 (20.2)	54.0 (19.4)
sCD163, ng /ml, mean (SD)	780.9 (368.4)	777.3 (385.1)
Trp, μg/mL, mean (SD)		0.16 (0.07)
Kyn, μg/mL, mean (SD)		0.009 (0.003)
Kyn/Trp ratio, mean (SD)		0.06 (0.034)
QUIN, μg/mL, mean (SD)		0.001 (0.001)
KA, ng/mL, mean (SD)		0.02 (0.02)

CD4^+^ count data was available for 39 participants (59%) in the overall cohort and 23 participants (48%) in the cohort with metabolite data only. Alcohol and smoking data were available for 99% of participants in both cohorts

### Differences in immune marker and metabolite levels across Tat/Vif variants

Participants were stratified based on Tat amino acid variants at position 24 (K: 14 vs. N: 13, *n* = 27), position 31 (C: 4 vs. S: 29, *n* = 33), position 57 (R: 5 vs. S: 26, *n* = 31), and position 68 (L: 17 vs. P: 15, *n* = 32), as well as Vif amino acid variants at position 17 (K: 25 vs. R: 23, *n* = 48) and position 31 (I: 13 vs. V: 30, *n* = 43). Using independent sample t-tests as well as χ2 tests, no significant differences were found in study characteristics (sex, age, locality, alcohol, BMI, and smoking) amongst the investigated groups.

Participants with the Tat N24 variant had higher levels of sCD163 compared to participants with the K24 variant (*p* = 0.04) (supplementary Fig. 1A). Moreover, sCD163 levels were significantly higher in participants with Tat S31 compared to participants with C31 (*p* < 0.001) (supplementary Fig. 2A). Nevertheless, following the application of a Bonferroni correction (*p* = 0.05/5 = 0.01), only higher levels of sCD163 remained statistically significant in participants with the S31 variant (*p* < 0.001). None of the remaining Tat amino acid variants (positions 24, 31, 57, and 68) displayed significant differences for any of the immune markers or metabolites investigated (supplementary Figs. 1–4). None of the immune markers or metabolites were significantly different between the Vif amino acid variants (supplementary Figs. 5–6).

**Fig. 2:**
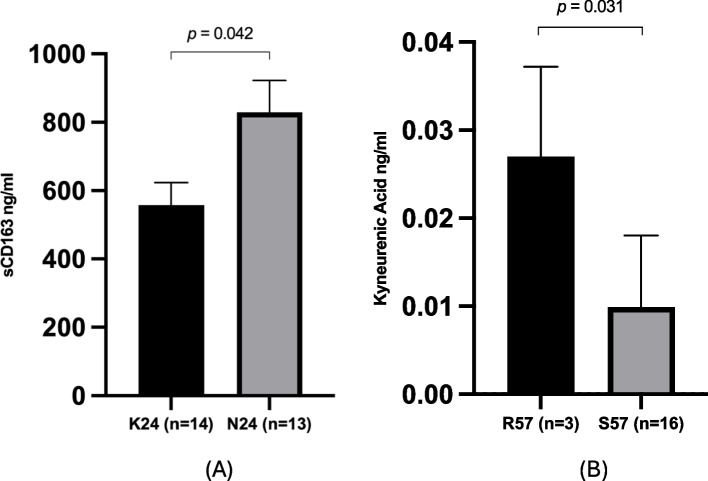
Significant findings for Tat position 24 and 57. (**A**) sCD163 levels were significantly higher in participants with the N24 amino acid variant in contrast to participants with the K24 amino acid variant (*p* = 0.042). KA levels were significantly higher in the participants with the R57 amino acid variant compared to participants with the S57 variant (*p* = 0.031). The bars depict the average protein concentrations across the diverse study groups and are articulated as mean values with standard error of the mean (SEM)

Furthermore, we aimed to determine whether our findings retained or gained significance after adjusting for covariates. After adjusting for locality, BMI, and sex using ANCOVA models for immune markers, it was found that among all Tat variants investigated, participants with the Tat N24 variant had higher levels of sCD163 (adj R^2^ = 0.048, *p* = 0.042) compared to those with the K24 variant (Fig. 2A). Upon adjusting for alcohol consumption and BMI to investigate metabolites, it was observed that participants with the Tat R57 group had higher levels of KA (adj R^2^ = 0.166,* p* = 0.031) compared to those in the S57 group (Fig. 2B). The full ANCOVA data for significant findings are presented in the Supplementary Tables 1 and 2. None of the metabolites/immune markers showed significant differences between the Vif amino acid variants and the remaining Tat amino acid variants after adjusting for covariates. Sample level data for immune markers and metabolites are presented in Supplementary Table 5 and 6.

### Relationship between Tat and Vif variants and immune marker/metabolite levels

**Fig. 3:**
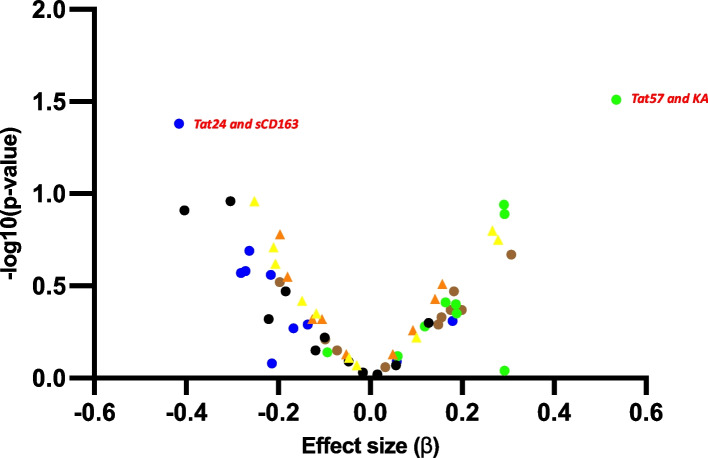
Volcano plot demonstrating the association between viral protein amino acid variations and immune marker/metabolite levels. The plot includes Tat amino acid position 24 (blue circles), position 31 (brown circles), position 57 (green circles), and position 68 (black circles). It also includes Vif amino acid positions 17 (orange triangles) and 31 (yellow triangles). Significant values, those with > -log10 1.3, are in red text

**Fig. 4:**
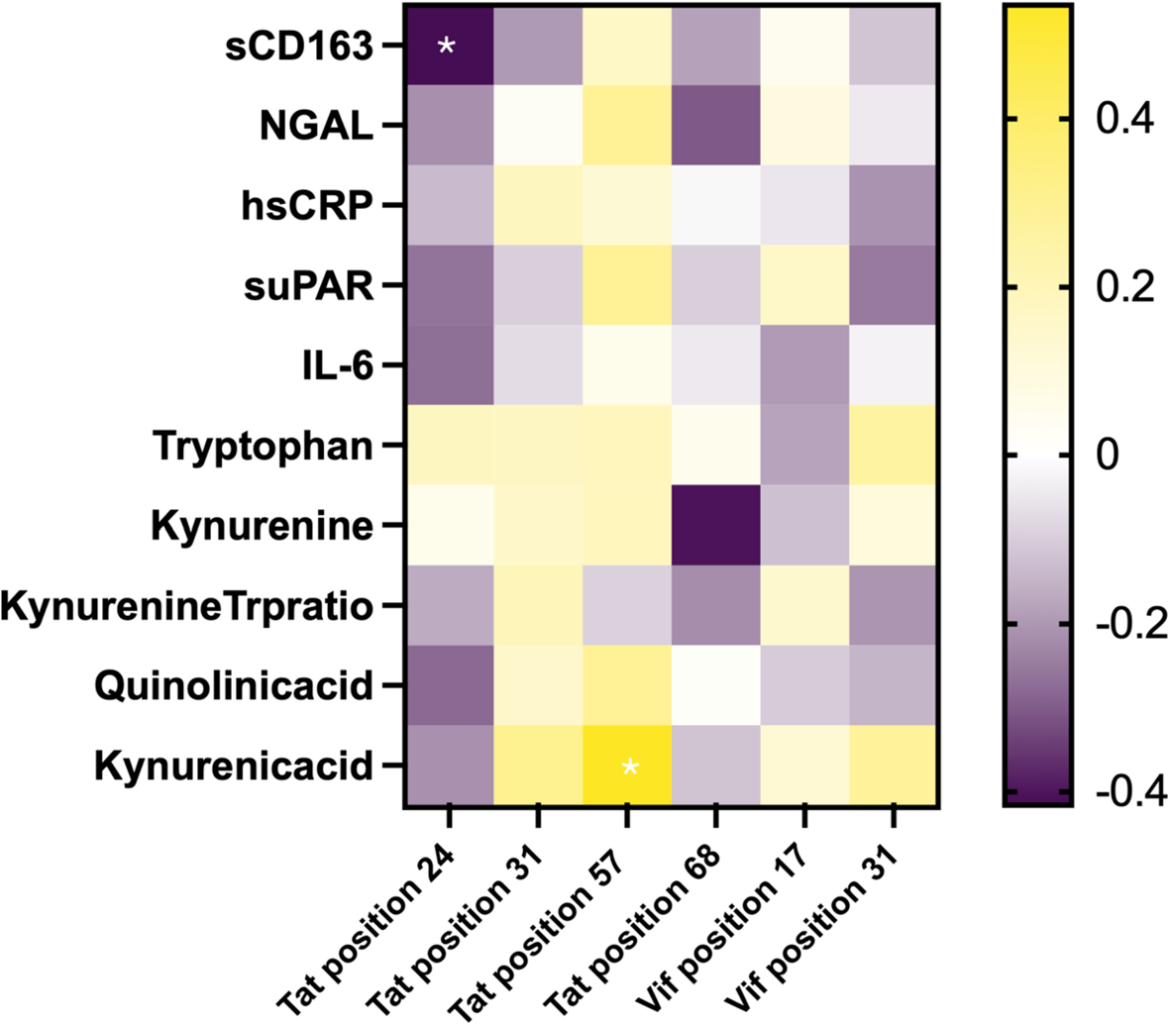
Heatmap representing the associations (normalized effect size β) between Tat/Vif amino acid positions and peripheral immune and metabolic markers. Significant associations with *p*-value < 0.05 are indicated by white asterisks

Additionally, we sought to assess the associations between Tat/Vif amino acid variations at specific positions and levels of immune markers/metabolites while controlling for covariates. To achieve this, we employed multiple regression analyses where covariates including locality, BMI, and sex were adjusted for in models focusing on immune markers, while alcohol use and BMI were adjusted for in models focusing on metabolites. The volcano plot (Fig. 3) illustrates the distribution of effect sizes and significance levels for the associations between amino acid positions in Tat and Vif and various immune markers and metabolites. Notably, significant associations were highlighted for Tat positions 24 and 57, highlighting their impact on specific immune markers and metabolic profiles. The heatmap (Fig. 4) further demonstrates these associations by mapping the normalized effect sizes for each Tat position against various immune markers/metabolites. Significant associations are marked with an asterisk, providing a clear visualization of the correlation patterns. This heatmap underscores the importance of certain Tat positions in influencing immune /metabolic changes, with Tat24 and Tat57 showing notable associations with sCD163 and KA, respectively. Among all Tat and Vif amino acid variants investigated, the amino acid variation of Tat at position 24 was found to be associated with sCD163 (adj R^2^ = 0.048, β = -0.416, *p* = 0.042). Similarly, the amino acid variation of Tat at position 57 was associated with KA (adj R^2^ = 0.166, β = 0.535, *p* = 0.031) (Fig. 3 and Fig. 4). The full regression data for significant findings are presented in Supplementary Tables 3A, 3B, 4A, and 4B.

## Discussion

In this study, we investigated the relationship between Tat and Vif amino acid sequence variations and peripheral immune markers, as well as Trp-Kyn metabolism, in a unique cohort of cART-naïve individuals from South Africa. This approach enabled us to analyse metabolic and immune profiles without the potential confounding effects of cART, which is known to influence these profiles in people with HIV [51, 52]. Several key findings emerged: (1) After adjusting for covariates, sCD163 and KA were higher in participants with Tat signatures N24 and R57, respectively and (2) amino acid variation at position 24 and 57 of Tat were associated with sCD163 and KA, respectively.

Firstly, we observed that participants with the N24 variant exhibited higher levels of sCD163 in contrast to participants with the K24 amino acid variant and amino acid variation at this position (between N24 and K24) was associated with sCD163. sCD163 is an immune activation marker often considered as an indicator of inflammation [53]. Dysregulated inflammation is often regarded as a negative clinical outcome, as higher levels of this specific immune markers have been linked to disease progression and neurocognitive impairment in individuals living with HIV [54, 55]. This finding is intriguing, particularly considering a recent study conducted among treatment naïve people with HIV in South Africa [11]. The study revealed that the Tat K24 variant was associated with a 2.08 times increased risk of neurocognitive impairment, 3.15 times higher proviral load, and a 69% lower absolute CD4 T-cell count compared to individuals without the signature. However, our findings suggest the N24 variant to be a higher risk amino acid compared to the K24 variant due its possible influence on inflammation. It is conceivable that position 24 of the Tat protein may play a functional role in HIV-1 pathogenesis overall, potentially influencing the structure–function relationship of the Tat protein.. This hypothesis aligns with observations in other regions of the Tat protein, such as the basic domain, where alterations of amino acids significantly affect Tat transactivation [26]. However, this previous study also investigated a small cohort; therefore, these findings warrant further validation in larger cohorts. Nonetheless, further investigation is necessary to elucidate the complete functional significance of variation at this position.

Furthermore, we observed that the Tat R57 variant exhibited higher levels of KA. KA has been linked to the pathology of several disorders [56]. Although our sample size for the amino acid position 57 split was small (R: 5 vs. S: 26, *n* = 31), and we cannot completely rule out the possibility of false positive findings in this group, the idea that the R57 amino acid variant may negatively influence clinical outcomes has been documented in clinical and in vitro models. In a previous study conducted within our group, albeit with a limited number of participants (R: 4 vs. S: 45, *n* = 49), lower TYMP and CCL2 levels were noted in the S57 group compared to the R57 group (*p* < 0.05) [20]. Similarly, in in vitro studies, the R57 variant was found to be associated with increased cellular uptake of Tat, transactivation (likely through enhanced interaction with TAR), and heightened inflammation [8, 27]. Here our results also showed that participants with the R57 signature had higher levels of KA, and amino acid variation at this position (between R57 and S57) was associated with KA. Hence, a consistent trend for the impact of the R57 variant is observed in the dysregulation of metabolism, indicating the necessity for larger cohort studies to further investigate this amino acid signature.

We also found that the Tat C31/C31S variant does not significantly influence the immune markers and metabolites investigated in this study. Similarly, in a previous study, amino acid variation at position 31 also did not show relevant differences in the levels of MCP-1/CCL2, matrix metalloproteinase (MMP)9, NGAL, transforming growth factor (TGF)-β1, TYMP, and vascular endothelial growth factor (VEGF) [20]. Despite extensive investigation of the C31 variant in cell culture studies, which showed that Tat C31 contributes to increased transmigration of cells into the CNS [57, 58], greater neurotoxicity to neuronal cells [59], and higher levels of inflammation [23, 24], these findings are not mirrored in clinical sample types or clinical outcomes. For instance, previous studies indicate that there are no significant disparities in cognitive performance between individuals exhibiting the C31S motif and those lacking the C31S substitution [60]. Likewise, there were no significant differences observed in either volumetric or diffusion indices between the Tat C31S and C31C groups [61]. This implies that the Tat C31S status might not serve as an adequate biomarker for adverse clinical outcomes, as the effects of mutations at this position could be concealed by other unexplored clinical factors.

A new variant identified in approximately half of our cohort was Tat P68, which also did not yield significant findings. It is noteworthy that the Vif R17 and V31 variant is found to be more commonly observed in South African subtype C Vif sequences in comparison to subtype C prevalent regions, such as India and Uganda [33]. Interestingly, a cell culture study suggests that K17 is considered more important for viral infectivity [28] and a The V31I mutation is known to induce cell cycle arrest [29]. However, within the context of this study, no significant findings were reported for the influence of these amino acid signatures. The precise roles of these Tat and Vif variants are yet to be fully explored.

Overall, it is evident from this study and many others that amino acid sequence variations of viral proteins may influence the structure–function relationships of these proteins, ultimately impacting the underlying mechanisms of HIV-1 pathogenesis. However, the extent to which these variations contribute to overall clinical outcomes in people with HIV requires further investigation and cannot be fully ascertained from this study alone. We acknowledge that certain signatures investigated in cell culture (without confounders) similarly reflect characteristics in clinical sample types, while others do not show this trend. This discrepancy may stem from the fact that in people with HIV, several other proteins may influence the levels of these markers, or there may be other confounding factors at play in clinical investigations. Additionally, it is worth considering that certain amino acids may not directly influence outcomes, but rather, their positioning in critical functional regions of key viral proteins may lead to changes in underlying mechanisms.

## Limitations

While recognizing the exploratory nature of this study, we acknowledge several limitations that are apparent, and therefore, caution should be exercised in interpreting our findings. Firstly, the number of participants per group was limited, and there were imbalances in group sizes in some cases, which may introduce the possibility of chance findings. Additionally, we may have lacked statistical power to detect other differences. Moreover, our investigation focused only on Tat and Vif, yet HIV involves multiple proteins that could influence the levels of metabolites and immune markers studied here. For instance, previous research by our group and others has linked certain signatures of other proteins like Vpr to immune markers [32]. Furthermore, there may be additional potential covariates that could have influenced our findings in the clinical sample. However, we have adjusted our analysis based on demographics and study characteristics that we have determined to have an influence. Another limitation of our study is related to the number of statistical tests performed and the multiple testing correction strategy employed. To identify covariates associated with our outcomes of interest, we conducted a correlation analysis. This approach allowed us to adjust for only those covariates that were significantly associated, thereby reducing the risk of overfitting the model. However, this method also has inherent limitations. To account for multiple comparisons, we applied the Bonferroni correction. While this conservative approach helps control the family-wise error rate, it also increases the likelihood of Type II errors, which means some true associations may not have been detected. The stringent nature of the Bonferroni correction can lead to an overly cautious interpretation of results, potentially overlooking meaningful findings. Lastly, we only investigated specific immune markers and metabolites, and there may be other markers more directly involved in pathways related to the amino acid changes of the proteins we investigated. Therefore, the exploratory nature of this study should be taken into consideration when interpreting the findings presented here.

## Conclusion

In a treatment-naïve cohort from South Africa subtype C, we investigated the associations between changes in amino acid sequences in Tat and Vif and specific immune and metabolic markers. After adjusting for covariates, sCD163 and KA were higher in participants with Tat signatures N24 and R57, respectively and amino acid variation at position 24 and 57 of Tat were associated with sCD163 and KA, respectively. Findings from this study highlight the potential influence of amino acid sequence variation of the Tat protein on inflammatory and metabolic pathways in people with HIV.

## Supplementary Information

Supplementary Material 1.

## Data Availability

The data supporting the findings of this study are available in the supplementary material of this article. The sequences are accessible in GenBank under the accession numbers OR621303-OR621349 for Tat and OR194556-OR194606 for Vif.

## References

[1] UNAIDS. In danger: UNAIDS global AIDS update 2022. Joint United Nations Programme on HIV/AIDS; 2022. Available from: https://www.unaids.org/en/resources/documents/2022/in-danger-global-aids-update.

[2] Taylor BS, Sobieszczyk ME, McCutchan FE, Hammer SM. The challenge of HIV-1 subtype diversity. N Engl J Med. 2008;358(15):1590–602 18403767 10.1056/NEJMra0706737 PMC2614444

[3] Yousaf MZ, Zia S, Babar ME, Ashfaq UA. The epidemic of HIV/AIDS in developing countries; the current scenario in Pakistan. Virol J. 2011;8:401. 21838892 10.1186/1743-422X-8-401 PMC3173394

[4] Asia LK, Jansen Van Vuren E, Williams ME. The influence of viral protein R amino acid substitutions on clinical outcomes in people living with HIV: A systematic review. Eur J Clin Invest. 2023;53(5):e13943. 10.1111/eci.13943 36579370

[5] Jin SW, Mwimanzi FM, Mann JK, Bwana MB, Lee GQ, Brumme CJ, Hunt PW, Martin JN, Bangsberg DR, Ndung’u T, et al. Variation in HIV-1 Nef function within and among viral subtypes reveals genetically separable antagonism of SERINC3 and SERINC5. PLoS Pathog. 2020;16(9): e1008813. 32925973 10.1371/journal.ppat.1008813 PMC7515180

[6] Frankel AD, Young JA. HIV-1: fifteen proteins and an RNA. Annu Rev Biochem. 1998;67:1–25. 9759480 10.1146/annurev.biochem.67.1.1

[7] Doolittle JM, Gomez SM. Structural similarity-based predictions of protein interactions between HIV-1 and Homo sapiens. Virology journal. 2010;7:1–15. 20426868 10.1186/1743-422X-7-82 PMC2877021

[8] Williams ME, Cloete R. Molecular Modeling of Subtype-Specific Tat Protein Signatures to Predict Tat-TAR Interactions That May Be Involved in HIV-Associated Neurocognitive Disorders. Front Microbiol. 2022;13: 866611. 35464972 10.3389/fmicb.2022.866611 PMC9021916

[9] Williams ME, Zulu SS, Stein DJ, Joska JA, Naudé PJW. Signatures of HIV-1 subtype B and C Tat proteins and their effects in the neuropathogenesis of HIV-associated neurocognitive impairments. Neurobiol Dis. 2020;136: 104701. 31837421 10.1016/j.nbd.2019.104701

[10] Demeulemeester J, Vets S, Schrijvers R, Madlala P, De Maeyer M, De Rijck J, Ndung’u T, Debyser Z, Gijsbers R. HIV-1 integrase variants retarget viral integration and are associated with disease progression in a chronic infection cohort. Cell Host Microbe. 2014;16(5):651–62. 25525795 10.1016/j.chom.2014.09.016

[11] Ruhanya V, Jacobs GB, Paul RH, Joska JA, Seedat S, Nyandoro G, Glashoff RH, Engelbrecht S. HIV-1 Subtype C Vpr Amino Acid Residue 45Y and Specific Conserved Fragments Are Associated with Neurocognitive Impairment and Markers of Viral Load. AIDS Res Hum Retroviruses. 2023;39(4):166–75. 36401355 10.1089/aid.2022.0022

[12] Ensoli B, Moretti S, Borsetti A, Maggiorella MT, Buttò S, Picconi O, Tripiciano A, Sgadari C, Monini P, Cafaro A. New insights into pathogenesis point to HIV-1 Tat as a key vaccine target. Arch Virol. 2021;166(11):2955–74. 34390393 10.1007/s00705-021-05158-z PMC8363864

[13] Ajasin D, Eugenin EA. HIV-1 Tat: Role in Bystander Toxicity. Front Cell Infect Microbiol. 2020;10:61. 32158701 10.3389/fcimb.2020.00061 PMC7052126

[14] Yang G, Xiong X. Viral infectivity factor: a novel therapeutic strategy to block HIV-1 replication. Mini Rev Med Chem. 2013;13(7):1047–55. 23621690 10.2174/1389557511313070008

[15] Schmitt K, Hill MS, Ruiz A, Culley N, Pinson DM, Wong SW, Stephens EB. Mutations in the highly conserved SLQYLA motif of Vif in a simian–human immunodeficiency virus result in a less pathogenic virus and are associated with G-to-A mutations in the viral genome. Virology. 2009;383(2):362–72. 19027134 10.1016/j.virol.2008.10.013 PMC4104693

[16] Alanazi A, Ivanov A, Kumari N, Lin X, Wang S, Kovalskyy D, Nekhai S. Targeting Tat–TAR RNA Interaction for HIV-1 Inhibition. Viruses. 2021;13(10):2004. 34696435 10.3390/v13102004 PMC8536978

[17] Li Y-L, Langley CA, Azumaya CM, Echeverria I, Chesarino NM, Emerman M, Cheng Y, Gross JD. The structural basis for HIV-1 Vif antagonism of human APOBEC3G. Nature. 2023;615(7953):728–33. 36754086 10.1038/s41586-023-05779-1 PMC10033410

[18] Mediouni S, Darque A, Baillat G, Ravaux I, Dhiver C, Tissot-Dupont H, Mokhtari M, Moreau H, Tamalet C, Brunet C, et al. Antiretroviral therapy does not block the secretion of the human immunodeficiency virus tat protein. Infect Disord Drug Targets. 2012;12(1):81–6. 22280310 10.2174/187152612798994939

[19] Fourati S, Malet I, Binka M, Boukobza S, Wirden M, Sayon S, Simon A, Katlama C, Simon V, Calvez V, et al. Partially active HIV-1 Vif alleles facilitate viral escape from specific antiretrovirals. AIDS. 2010;24(15):2313–21. 20729708 10.1097/QAD.0b013e32833e515a PMC12145881

[20] Williams ME, Ruhanya V, Paul RH, Ipser JC, Stein DJ, Joska JA, Naudé PJW. An investigation of the HIV Tat C31S and R57S mutation on peripheral immune marker levels in South African participants: A pilot study. J Med Virol. 2022;94(7):2936–8. 35285039 10.1002/jmv.27720

[21] Ruhanya V, Jacobs GB, Paul RH, Joska JA, Seedat S, Nyandoro G, Glashoff RH, Engelbrecht S. HIV-1 subtype C Tat exon-1 amino acid residue 24K is a signature for neurocognitive impairment. J Neurovirol. 2022;28(3):392–403. 35394614 10.1007/s13365-022-01073-4

[22] Gandhi N, Saiyed Z, Thangavel S, Rodriguez J, Rao K, Nair MP. Differential effects of HIV type 1 clade B and clade C Tat protein on expression of proinflammatory and antiinflammatory cytokines by primary monocytes. AIDS Res Hum Retroviruses. 2009;25(7):691–9. 19621989 10.1089/aid.2008.0299 PMC2853861

[23] Campbell GR, Watkins JD, Singh KK, Loret EP, Spector SA. Human immunodeficiency virus type 1 subtype C Tat fails to induce intracellular calcium flux and induces reduced tumor necrosis factor production from monocytes. J Virol. 2007;81(11):5919–28. 17376903 10.1128/JVI.01938-06 PMC1900281

[24] Wong JK, Campbell GR, Spector SA. Differential induction of interleukin-10 in monocytes by HIV-1 clade B and clade C Tat proteins. J Biol Chem. 2010;285(24):18319–25. 20378550 10.1074/jbc.M110.120840 PMC2881757

[25] Gotora PT, Brown K, Martin DR, van der Sluis R, Cloete R, Williams ME. Impact of subtype C-specific amino acid variants on HIV-1 Tat-TAR interaction: insights from molecular modelling and dynamics. Virology Journal. 2024;21(1):144. 38918875 10.1186/s12985-024-02419-6 PMC11202254

[26] Gotora PT, van der Sluis R, Williams ME. HIV-1 Tat amino acid residues that influence Tat-TAR binding affinity: a scoping review. BMC Infect Dis. 2023;23(1):164. 36932337 10.1186/s12879-023-08123-0 PMC10020771

[27] Ruiz AP, Ajasin DO, Ramasamy S, DesMarais V, Eugenin EA, Prasad VR. A Naturally Occurring Polymorphism in the HIV-1 Tat Basic Domain Inhibits Uptake by Bystander Cells and Leads to Reduced Neuroinflammation. Sci Rep. 2019;9(1):3308. 30824746 10.1038/s41598-019-39531-5 PMC6397180

[28] Iwabu Y, Kinomoto M, Tatsumi M, Fujita H, Shimura M, Tanaka Y, Ishizaka Y, Nolan D, Mallal S, Sata T, et al. Differential anti-APOBEC3G activity of HIV-1 Vif proteins derived from different subtypes. J Biol Chem. 2010;285(46):35350–8. 20833716 10.1074/jbc.M110.173286 PMC2975159

[29] Marelli S, Williamson JC, Protasio AV, Naamati A, Greenwood EJ, Deane JE, Lehner PJ, Matheson NJ. Antagonism of PP2A is an independent and conserved function of HIV-1 Vif and causes cell cycle arrest. Elife. 2020;9:e53036. 10.7554/eLife.53036 PMC7920553 32292164

[30] Salamango DJ, Ikeda T, Moghadasi SA, Wang J, McCann JL, Serebrenik AA, Ebrahimi D, Jarvis MC, Brown WL, Harris RS. HIV-1 Vif Triggers Cell Cycle Arrest by Degrading Cellular PPP2R5 Phospho-regulators. Cell Rep. 2019;29(5):1057-1065.e1054. 31665623 10.1016/j.celrep.2019.09.057 PMC6903395

[31] Asia LK, Van Vuren EJ, Kruger IM, Williams ME. A Pilot Investigation of the Association Between Vpr Amino Acid Substitutions and Peripheral Immune Marker Levels in People With Human Immunodeficiency Virus: Implications for Neurocognitive Impairment. Open Forum Infect Dis. 2024;11(3):ofae111. 38524224 10.1093/ofid/ofae111 PMC10960601

[32] Asia LK, Van Vuren EJ, Lindeque Z, Williams ME. A pilot investigation of the association between HIV-1 Vpr amino acid sequence diversity and the tryptophan-kynurenine pathway as a potential mechanism for neurocognitive impairment. Virol J. 2024;21(1):47. 38395987 10.1186/s12985-024-02313-1 PMC10893664

[33] Williams ME. HIV-1 Vif protein sequence variations in South African people living with HIV and their influence on Vif-APOBEC3G interaction. Eur J Clin Microbiol Infect Dis. 2024;43(2):325–38. 38072879 10.1007/s10096-023-04728-0 PMC10821834

[34] Gartner MJ, Roche M, Churchill MJ, Gorry PR, Flynn JK. Understanding the mechanisms driving the spread of subtype C HIV-1. EBioMedicine. 2020;53: 102682. 32114391 10.1016/j.ebiom.2020.102682 PMC7047180

[35] Ronsard L, Raja R, Panwar V, Saini S, Mohankumar K, Sridharan S, Padmapriya R, Chaudhuri S, Ramachandran VG, Banerjea AC. Genetic and functional characterization of HIV-1 Vif on APOBEC3G degradation: First report of emergence of B/C recombinants from North India. Sci Rep. 2015;5(1):15438. 26494109 10.1038/srep15438 PMC4616021

[36] Lv T, Cao W, Li T. HIV-Related Immune Activation and Inflammation: Current Understanding and Strategies. J Immunol Res. 2021;2021:7316456. 34631899 10.1155/2021/7316456 PMC8494587

[37] Peltenburg NC, Schoeman JC, Hou J, Mora F, Harms AC, Lowe SH, Bierau J, Bakker JA, Verbon A, Hankemeier T, et al. Persistent metabolic changes in HIV-infected patients during the first year of combination antiretroviral therapy. Sci Rep. 2018;8(1):16947. 30446683 10.1038/s41598-018-35271-0 PMC6240055

[38] Routy JP, Mehraj V, Vyboh K, Cao W, Kema I, Jenabian MA. Clinical Relevance of Kynurenine Pathway in HIV/AIDS: An Immune Checkpoint at the Crossroads of Metabolism and Inflammation. AIDS Rev. 2015;17(2):96–106. 26035167

[39] Huengsberg M, Winer JB, Gompels M, Round R, Ross J, Shahmanesh M. Serum kynurenine-to-tryptophan ratio increases with progressive disease in HIV-infected patients. Clin Chem. 1998;44(4):858–62. 9554499 10.1093/clinchem/44.4.858

[40] Sultana S, Elengickal A, Bensreti H. Belin de Chantemèle E, McGee-Lawrence ME, Hamrick MW: The kynurenine pathway in HIV, frailty and inflammaging. Front Immunol. 2023;14:1244622. 37744363 10.3389/fimmu.2023.1244622 PMC10514395

[41] Haroon E, Welle JR, Woolwine BJ, Goldsmith DR, Baer W, Patel T, Felger JC, Miller AH. Associations among peripheral and central kynurenine pathway metabolites and inflammation in depression. Neuropsychopharmacology. 2020;45(6):998–1007. 31940661 10.1038/s41386-020-0607-1 PMC7162907

[42] Teo K, Chow CK, Vaz M, Rangarajan S, Yusuf S. The Prospective Urban Rural Epidemiology (PURE) study: Examining the impact of societal influences on chronic noncommunicable diseases in low-, middle-, and high-income countries. Am Heart J. 2009;158(1):1-7.e1. 19540385 10.1016/j.ahj.2009.04.019

[43] Badawy AA, Guillemin G. The Plasma [Kynurenine]/[Tryptophan] Ratio and Indoleamine 2,3-Dioxygenase: Time for Appraisal. Int J Tryptophan Res. 2019;12:1178646919868978. 31488951 10.1177/1178646919868978 PMC6710706

[44] Mabhida SE, McHiza ZJ, Mokgalaboni K, Hanser S, Choshi J, Mokoena H, Ziqubu K, Masilela C, Nkambule BB, Ndwandwe DE, et al. High-sensitivity C-reactive protein among people living with HIV on highly active antiretroviral therapy: a systemic review and meta-analysis. BMC Infect Dis. 2024;24(1):160. 38308222 10.1186/s12879-024-09050-4 PMC10838000

[45] Knudsen TB, Ertner G, Petersen J, Møller HJ, Moestrup SK, Eugen-Olsen J, Kronborg G, Benfield T. Plasma Soluble CD163 Level Independently Predicts All-Cause Mortality in HIV-1–Infected Individuals. J Infect Dis. 2016;214(8):1198–204. 27354366 10.1093/infdis/jiw263

[46] Borges ÁH, O’Connor JL, Phillips AN, Rönsholt FF, Pett S, Vjecha MJ, French MA, Lundgren JD. Factors Associated With Plasma IL-6 Levels During HIV Infection. J Infect Dis. 2015;212(4):585–95. 25722296 10.1093/infdis/jiv123 PMC4598808

[47] Hoenigl M, Moser CB, Funderburg N, Bosch R, Kantor A, Zhang Y, Eugen-Olsen J, Finkelman M, Reiser J, Landay A, et al. Soluble Urokinase Plasminogen Activator Receptor Is Predictive of Non-AIDS Events During Antiretroviral Therapy-mediated Viral Suppression. Clin Infect Dis. 2019;69(4):676–86. 30418519 10.1093/cid/ciy966 PMC6669298

[48] Williams ME, Ipser JC, Stein DJ, Joska JA, Naudé PJW. The Association of Immune Markers with Cognitive Performance in South African HIV-Positive Patients. J Neuroimmune Pharmacol. 2019;14(4):679–87. 31388873 10.1007/s11481-019-09870-1

[49] Williams ME, Joska JA, Amod AR, Paul RH, Stein DJ, Ipser JC, Naudé PJ. The association of peripheral immune markers with brain cortical thickness and surface area in South African people living with HIV. J Neurovirol. 2020;26:908–19. 32661895 10.1007/s13365-020-00873-w

[50] Gasteiger E, Gattiker A, Hoogland C, Ivanyi I, Appel RD, Bairoch A. ExPASy: The proteomics server for in-depth protein knowledge and analysis. Nucleic Acids Res. 2003;31(13):3784–8. 12824418 10.1093/nar/gkg563 PMC168970

[51] Keegan MR, Winston A, Higgs C, Fuchs D, Boasso A, Nelson M. Tryptophan metabolism and its relationship with central nervous system toxicity in people living with HIV switching from efavirenz to dolutegravir. J Neurovirol. 2019;25(1):85–90. 30478800 10.1007/s13365-018-0688-3 PMC6416362

[52] Osuji FN, Onyenekwe CC, Ahaneku JE, Ukibe NR. The effects of highly active antiretroviral therapy on the serum levels of pro-inflammatory and anti-inflammatory cytokines in HIV infected subjects. J Biomed Sci. 2018;25(1):88. 30501642 10.1186/s12929-018-0490-9 PMC6276218

[53] Castley A, Williams L, James I, Guelfi G, Berry C, Nolan D. Plasma CXCL10, sCD163 and sCD14 Levels Have Distinct Associations with Antiretroviral Treatment and Cardiovascular Disease Risk Factors. PLoS ONE. 2016;11(6):e0158169–e0158169. 27355513 10.1371/journal.pone.0158169 PMC4927121

[54] Generoso M, Álvarez P, Kravietz A, Mwamzuka M, Marshed F, Ahmed A, Khaitan A. High soluble CD163 levels correlate with disease progression and inflammation in Kenyan children with perinatal HIV-infection. AIDS. 2020;34(1):33–8. 31567161 10.1097/QAD.0000000000002378

[55] Borges ÁH, O’Connor JL, Phillips AN, Neaton JD, Grund B, Neuhaus J, Vjecha MJ, Calmy A, Koelsch KK, Lundgren JD. Interleukin 6 Is a Stronger Predictor of Clinical Events Than High-Sensitivity C-Reactive Protein or D-Dimer During HIV Infection. J Infect Dis. 2016;214(3):408–16. 27132283 10.1093/infdis/jiw173 PMC4936649

[56] Wirthgen E, Hoeflich A, Rebl A, Günther J. Kynurenic Acid: The Janus-Faced Role of an Immunomodulatory Tryptophan Metabolite and Its Link to Pathological Conditions. Front Immunol. 1957;2017:8. 10.3389/fimmu.2017.01957 PMC5770815 29379504

[57] Ranga U, Shankarappa R, Siddappa NB, Ramakrishna L, Nagendran R, Mahalingam M, Mahadevan A, Jayasuryan N, Satishchandra P, Shankar SK, et al. Tat protein of human immunodeficiency virus type 1 subtype C strains is a defective chemokine. J Virol. 2004;78(5):2586–90. 14963162 10.1128/JVI.78.5.2586-2590.2004 PMC369202

[58] Rao VR, Neogi U, Talboom JS, Padilla L, Rahman M, Fritz-French C, Gonzalez-Ramirez S, Verma A, Wood C, Ruprecht RM, et al. Clade C HIV-1 isolates circulating in Southern Africa exhibit a greater frequency of dicysteine motif-containing Tat variants than those in Southeast Asia and cause increased neurovirulence. Retrovirology. 2013;10(1):61. 23758766 10.1186/1742-4690-10-61 PMC3686704

[59] Li W, Huang Y, Reid R, Steiner J, Malpica-Llanos T, Darden TA, Shankar SK, Mahadevan A, Satishchandra P, Nath A. NMDA receptor activation by HIV-Tat protein is clade dependent. J Neurosci. 2008;28(47):12190–8. 19020013 10.1523/JNEUROSCI.3019-08.2008 PMC6671692

[60] Paul RH, Joska JA, Woods C, Seedat S, Engelbrecht S, Hoare J, Heaps J, Valcour V, Ances B, Baker LM, et al. Impact of the HIV Tat C30C31S dicysteine substitution on neuropsychological function in patients with clade C disease. J Neurovirol. 2014;20(6):627–35. 25366660 10.1007/s13365-014-0293-z PMC4268069

[61] Paul RH, Phillips S, Hoare J, Laidlaw DH, Cabeen R, Olbricht GR, Su Y, Stein DJ, Engelbrecht S, Seedat S, et al. Neuroimaging abnormalities in clade C HIV are independent of Tat genetic diversity. J Neurovirol. 2017;23(2):319–28. 27913960 10.1007/s13365-016-0503-y PMC5334278

