# Supplementary material for: The association between HIV-1 Tat and Vif amino acid sequence variation, inflammation and Trp-Kyn metabolism: an exploratory investigation
Source: BMC Infect Dis. 2024 Sep 9;24:943. doi: 10.1186/s12879-024-09874-0 (PMC11385500; doi:10.1186/s12879-024-09874-0)
Supplement: Supplementary file 1 — Supplementary Material 1. [file 12879_2024_9874_MOESM1_ESM.docx]

**Supplementary Figures**


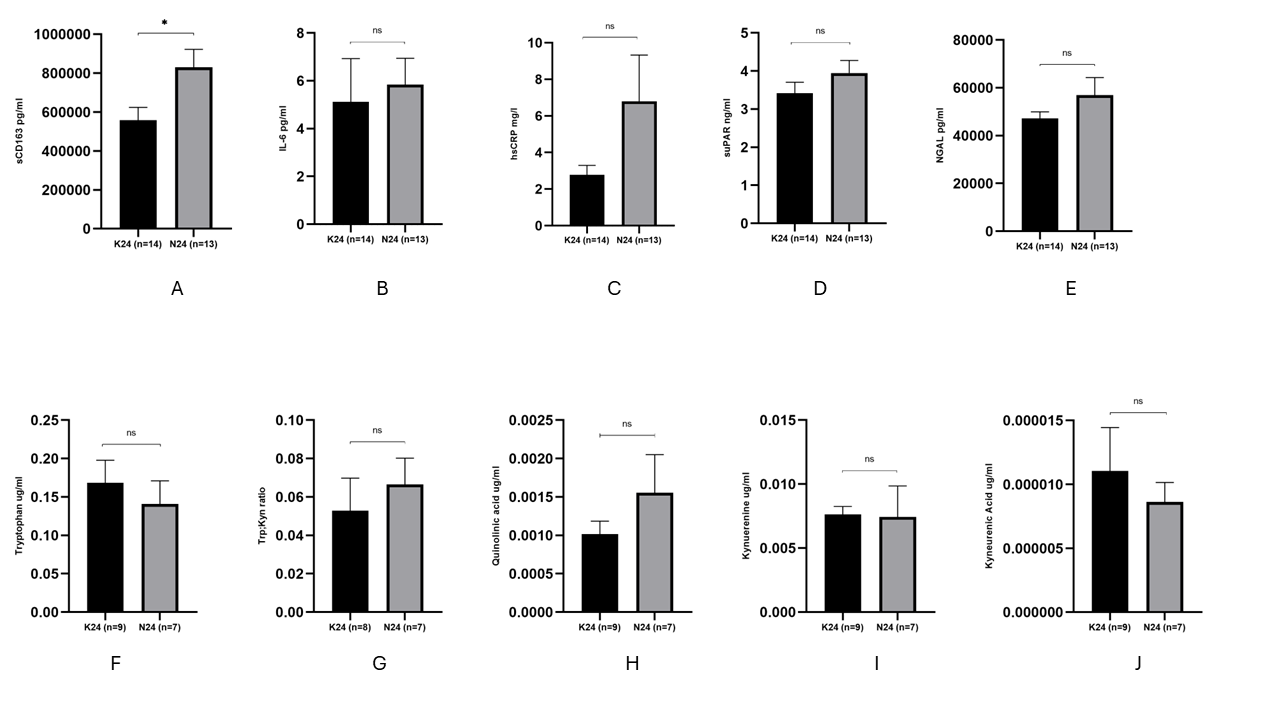


**Figure 1:** Differences in peripheral immune marker (A-E) and metabolite levels (E-I) between Tat K24 and N24 amino acid variants. The bars indicate mean protein concentrations in the different study groups and are expressed as mean ± standard error of the mean (SEM).


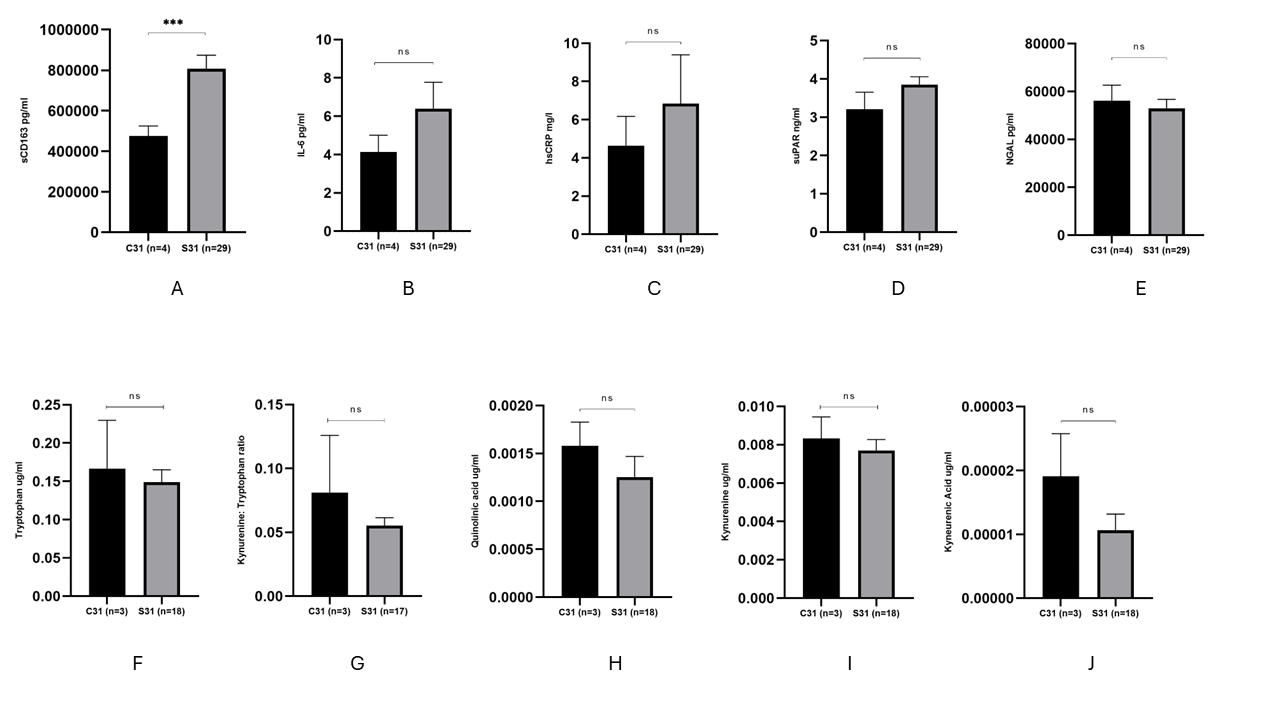


**Figure 2:** Differences in peripheral immune marker (A-E) and metabolite levels (F-J) between Tat C31 and S31 amino acid variants. The bars indicate mean protein concentrations in the different study groups and are expressed as mean ± standard error of the mean (SEM).
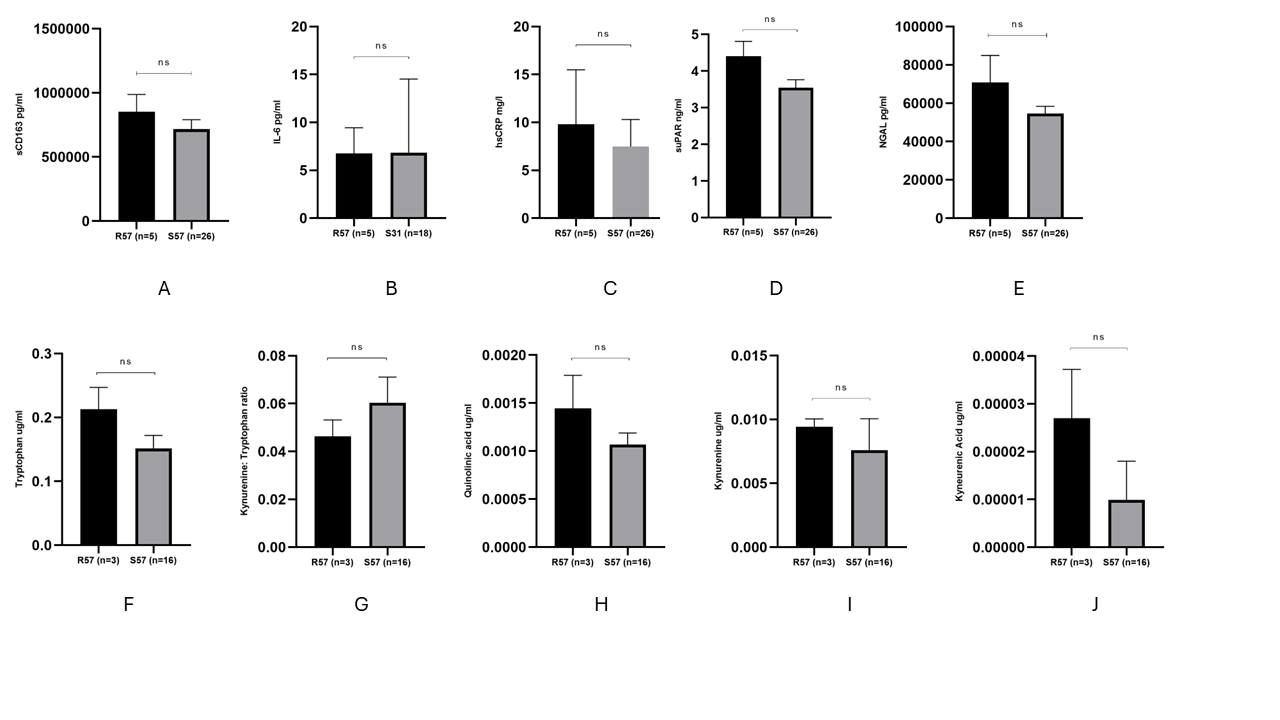
**Figure 3:** Differences in peripheral immune marker (A-E) and metabolite levels (F-J) between Tat R57 and S57 amino acid variants. The bars indicate mean protein concentrations in the different study groups and are expressed as mean ± standard error of the mean (SEM).


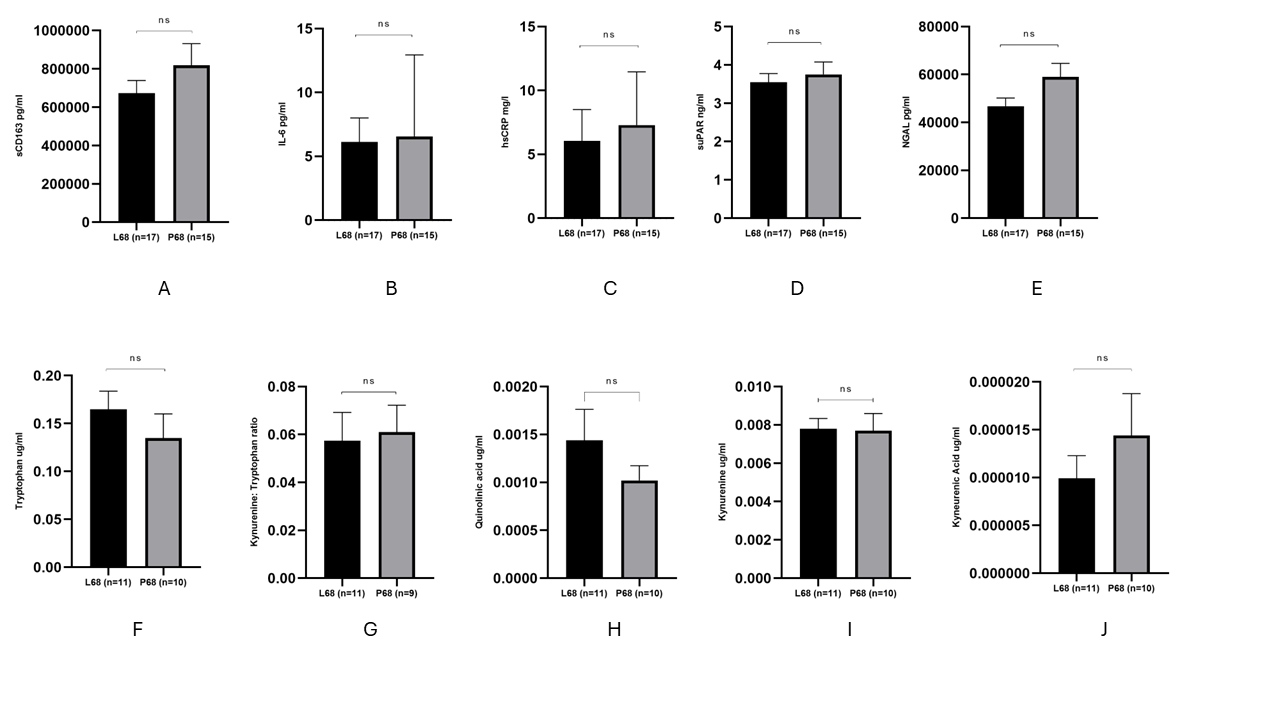
**Figure 4:** Differences in peripheral immune marker (A-E) and metabolite levels (F-J) between Tat L68 and P68 amino acid variants. The bars indicate mean protein concentrations in the different study groups and are expressed as mean ± standard error of the mean (SEM).


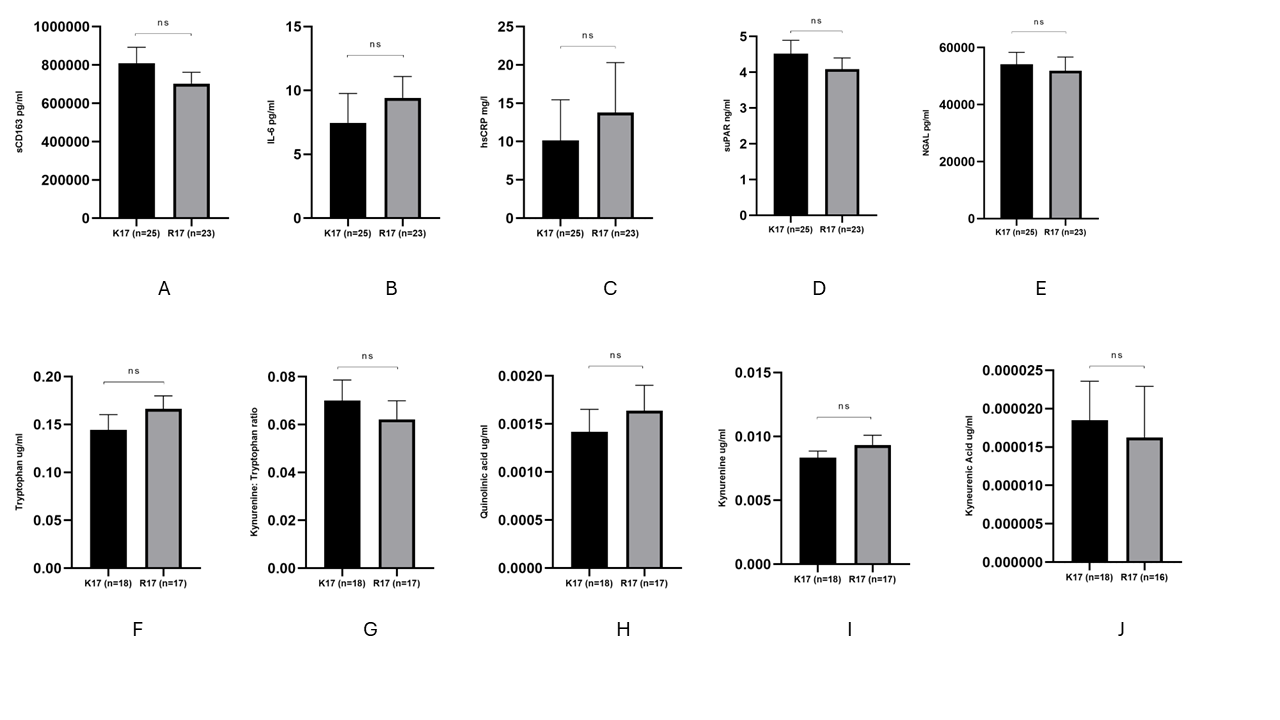


**Figure 5:** Differences in peripheral immune marker (A-E) and metabolite levels (F-J) between Vif K17 and R17 amino acid variants. The bars indicate mean protein concentrations in the different study groups and are expressed as mean ± standard error of the mean (SEM).


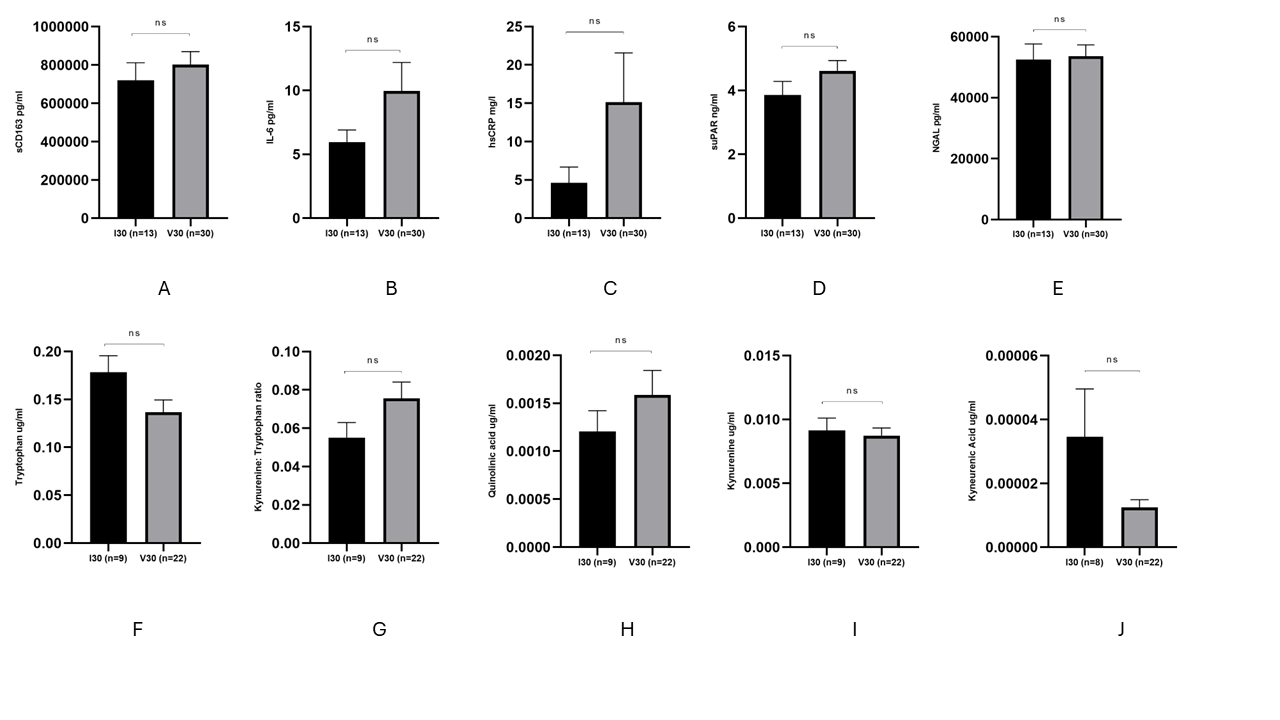


**Figure 6:** Differences in peripheral immune marker (A-E) and metabolite levels (F-J) between Vif I30 and V30 amino acid variants. The bars indicate mean protein concentrations in the different study groups and are expressed as mean ± standard error of the mean (SEM).

**Supplementary Tables**

**Table 1: Full ANCOVA data for sCD163 and Tat position 24**

| **Tests of Between-Subjects Effects** | | | | | | |
| --- | --- | --- | --- | --- | --- | --- |
| Dependent Variable: logscd163 | | | | | | |
| Source | Type III Sum of Squares | df | Mean Square | F | Sig. | Partial Eta Squared |
| Corrected Model | .228^a^ | 4 | .057 | 1.330 | .290 | .195 |
| Intercept | 32.884 | 1 | 32.884 | 767.689 | <.001 | .972 |
| Localitycode | 2.926E-5 | 1 | 2.926E-5 | .001 | .979 | .000 |
| Gendercode | .015 | 1 | .015 | .350 | .560 | .016 |
| BMI | .037 | 1 | .037 | .860 | .364 | .038 |
| Pos24 | .201 | 1 | .201 | 4.682 | .042 | .175 |
| Error | .942 | 22 | .043 |  |  |  |
| Total | 905.638 | 27 |  |  |  |  |
| Corrected Total | 1.170 | 26 |  |  |  |  |
| 1. R Squared = .195 (Adjusted R Squared = .048) | | | | | | |

**Table 2: Full ANCOVA data for Kynurenic acid and Tat position 57**

| **Tests of Between-Subjects Effects** | | | | | |
| --- | --- | --- | --- | --- | --- |
| Dependent Variable: logka_1 | | | | | |
| Source | Type III Sum of Squares | df | Mean Square | F | Sig. |
| Corrected Model | 5.644^a^ | 3 | 1.881 | 2.194 | .131 |
| Intercept | 95.946 | 1 | 95.946 | 111.905 | <.001 |
| Alc_cons2010 | .126 | 1 | .126 | .147 | .707 |
| BMI | 1.983 | 1 | 1.983 | 2.312 | .149 |
| Pos57 | 4.863 | 1 | 4.863 | 5.672 | .031 |
| Error | 12.861 | 15 | .857 |  |  |
| Total | 2620.452 | 19 |  |  |  |
| Corrected Total | 18.505 | 18 |  |  |  |
| a. R Squared = .305 (Adjusted R Squared = .166) | | | | | |

**Table 3A:** Multiple regression analysis indicating model summary for association between sCD163 and Tat position 24

| **Model Summary^b^** | | | | | |
| --- | --- | --- | --- | --- | --- |
| Model | R | R Square | Adjusted R Square | Std. Error of the Estimate | Durbin-Watson |
| 1 | .441^a^ | .195 | .048 | .20697 | 1.988 |
| a. Predictors: (Constant), BMI, Pos24, Locality code, Gender code | | | | | |
| b. Dependent Variable: logscd163 | | | | | |

**Table 3B:** Multiple regression analysis indicating coefficients for association between sCD163 and Tat position 24

| **Coefficients^a^** | | | | | | |
| --- | --- | --- | --- | --- | --- | --- |
| Model | | Unstandardized Coefficients | | Standardized Coefficients | t | Sig. |
|  |  | B | Std. Error | Beta |  |  |
| 1 | (Constant) | 5.699 | .204 |  | 27.918 | <.001 |
|  | Pos24 | -.173 | .080 | -.416 | -2.164 | .042 |
|  | Localitycode | .002 | .082 | .005 | .026 | .979 |
|  | Gendercode | .056 | .095 | .124 | .592 | .560 |
|  | BMI | .007 | .007 | .197 | .927 | .364 |
| a. Dependent Variable: logscd163 | | | | | | |

**Table 4A:** Multiple regression analysis indicating model summary for association between Kynurenic acid and Tat position 57

| **Model Summary^b^** | | | | | |
| --- | --- | --- | --- | --- | --- |
| Model | R | R Square | Adjusted R Square | Std. Error of the Estimate | Durbin-Watson |
| 1 | .552^a^ | .305 | .166 | .92595 | 2.854 |
| a. Predictors: (Constant), BMI, Alc_cons2010, Pos57 | | | | | |
| b. Dependent Variable: logka_1 | | | | | |

**Table 4B:** Multiple regression analysis indicating coefficients for association between Kynurenic acid and Tat position 57

| **Coefficients^a^** | | | | | | |
| --- | --- | --- | --- | --- | --- | --- |
| Model | | Unstandardized Coefficients | | Standardized Coefficients | t | Sig. |
|  |  | B | Std. Error | Beta |  |  |
| 1 | (Constant) | -10.753 | .857 |  | -12.541 | <.001 |
|  | Pos57 | 1.447 | .607 | .535 | 2.382 | .031 |
|  | Alc_cons2010 | .166 | .432 | .083 | .384 | .707 |
|  | BMI | -.051 | .034 | -.341 | -1.521 | .149 |
| a. Dependent Variable: logka_1 | | | | | | |

**Table 5:** Immune marker levels per participant

| **Participant** | **hsCRP (mg/L)** | **IL6 (pg/mL)** | **suPAR (ng/mL)** | **nGAL (ng/ml)** | **sCD163 (pg/ml)** |
| --- | --- | --- | --- | --- | --- |
| 1 | 1.2000 | 13.470000000000000 | 4.128581411600280 | 71494.410602935600 | 902461.04881909900 |
| 2 | 1.0600 | 7.280000000000000 | 4.173702519923780 | 49849.434518332900 | 795485.00994408700 |
| 3 | 9.8600 | 5.710000000000000 | 5.263618742924960 | 73245.288558108400 | 1216965.76060807000 |
| 4 | 2.1800 | 7.770000000000000 | 4.834907714089440 | 49505.675446645100 | 1129762.65127171000 |
| 5 | 2.8200 | 2.040000000000000 | 3.858399259519660 | 72838.347336349300 | 1040931.65651794000 |
| 6 | 64.6800 | 7.710000000000000 | 4.816845072926870 | 75770.568676165500 | 1111291.87633511000 |
| 7 | 41.5700 | 35.210000000000000 | 4.297244985729520 | 66389.727719376400 | 609443.68051065600 |
| 8 | 0.3300 | 4.420000000000000 | 3.394192077889190 | 30240.742058559300 | 381427.86355321800 |
| 9 | 13.7500 | 20.560000000000000 | 5.705061044462570 | 38192.749839369900 | 1250414.08961965000 |
| 10 | 148.0000 | 18.700000000000000 | 4.705965776974600 | 88136.342416539500 | 692598.59386866200 |
| 11 | 15.9100 | 7.920000000000000 | 4.433230469079750 | 41795.551875541300 | 740480.90232080400 |
| 12 | 0.5500 | 6.960000000000000 | 2.777137184677670 | 52299.847453227100 | 1142126.49747042000 |
| 13 | 1.4200 | 2.278000000000000 | 3.173871068203050 | 32226.567688233300 | 556700.10582889400 |
| 14 | 14.6500 | 8.390000000000000 | 4.884373088857050 | 60295.339702132900 | 973503.02432459200 |
| 15 | 0.3700 | 6.230000000000000 | 6.761566092501640 | 32199.999925137400 | 1419920.90626516000 |
| 16 | 2.5900 | 13.370000000000000 | 7.305743937134060 | 69557.622129851600 | 1612041.80973156000 |
| 17 | 133.9700 | 60.150000000000000 | 10.766469669505200 | 84317.260718016200 | 1054327.87092406000 |
| 18 | 0.6700 | 1.980000000000000 | 2.492577030127230 | 33299.027051548200 | 648761.35670075300 |
| 19 | 22.3700 | 13.710000000000000 | 5.334355673170840 | 26154.281516221200 | 913698.31309109200 |
| 20 | 6.1800 | 4.960000000000000 | 3.830894119700890 | 31042.645152683000 | 1314120.78638798000 |
| 21 | 0.4500 | 7.100000000000000 | 2.056649887980450 | 33842.453633130500 | 1267306.05505969000 |
| 22 | 0.5500 | 3.030000000000000 | 3.800197852716110 | 30782.756153108800 | 696866.10389325800 |
| 23 | 1.1900 | 1.930000000000000 | 4.328173644854380 | 33651.704327893000 | 970754.86765804200 |
| 24 | 16.6700 | 22.550000000000000 | 8.593880529578310 | 27703.657630287800 | 491328.53469508300 |
| 25 | 4.6500 | 6.820000000000000 | 2.274624107409200 | 70690.164964800800 | 459854.83682756600 |
| 26 | 3.9700 | 6.230000000000000 | 3.099198584791870 | 109030.085163274000 | 442203.77650410700 |
| 27 | 3.0500 | 4.250000000000000 | 4.332091452464550 | 56653.310342617400 | 290804.30186577200 |
| 28 | 8.5396 | 7.050000000000000 | 4.323688057069320 | 80328.955591322900 | 647004.07016182100 |
| 29 | 2.4800 | 4.410000000000000 | 3.259697539751770 | 62242.807739389400 | 916274.38552999000 |
| 30 | 2.0499 | 2.760000000000000 | 2.622372539362260 | 48556.346807383800 | 252137.84462658700 |
| 31 | 13.9300 | 2.690000000000000 | 4.029845859583230 | 76293.166587423200 | 340573.93335698500 |
| 32 | 1.8400 | 2.930000000000000 | 2.417955140940370 | 37637.690853457700 | 414250.46268675200 |
| 33 | 5.0100 | 4.030000000000000 | 6.345006251152880 | 29754.280481329300 | 652764.47336128200 |
| 34 | 2.8000 | 16.420000000000000 | 5.139673490541590 | 30705.922995120800 | 1092527.98657458000 |
| 35 | 18.3700 | 13.100000000000000 | 3.721632846186630 | 58106.479091725600 | 474900.19499593700 |
| 36 | 1.6000 | 11.730000000000000 | 8.009964952552720 | 57897.050138613500 | 1400732.16038344000 |
| 37 | 0.7200 | 5.710000000000000 | 4.443926133918140 | 38921.107102657100 | 1407645.86503056000 |
| 38 | 0.4000 | 2.360000000000000 | 4.067292588657800 | 28093.653497929300 | 708213.56919873800 |
| 39 | 3.3000 | 7.110000000000000 | 4.484887557572550 | 57065.461653387300 | 918612.62346162200 |
| 40 | 7.6600 | 3.950000000000000 | 2.732514273487910 | 34985.249710569200 | 171275.84769663200 |
| 41 | 6.1500 | 3.140000000000000 | 2.191189702937090 | 43040.019818878500 | 310458.75703724400 |
| 42 | 0.4500 | 1.770000000000000 | 3.935805417503850 | 47642.506735663600 | 1122059.12211932000 |
| 43 | 6.5200 | 7.160000000000000 | 4.231523561306550 | 35166.566135021000 | 1238385.48733512000 |
| 44 | 18.7300 | 7.810000000000000 | 3.809050707297890 | 71583.410817699300 | 738751.88266292500 |
| 45 | 3.3400 | 4.360000000000000 | 3.475712471701120 | 29045.887288633400 | 855328.02825572300 |
| 46 | 1.0900 | 4.330000000000000 | 3.462757727351040 | 81616.759897095400 | 558377.33359269100 |
| 47 | 0.3800 | 4.410000000000000 | 4.159076907300900 | 55768.536060614800 | 1409130.25697914000 |
| 48 | 0.7500 | 5.320000000000000 | 6.884069603053990 | 76027.965668267000 | 1593379.18639967000 |
| 49 | 8.9400 | 4.780000000000000 | 3.540325390656410 | 74844.990296279200 | 610817.59704231700 |
| 50 | 0.7100 | 3.560000000000000 | 2.904179523041830 | 47771.403279816900 | 769306.29203188300 |
| 51 | 31.7400 | 16.700000000000000 | 5.249862983960230 | 118731.698075007000 | 524585.81330044400 |
| 52 | 8.0100 | 10.010000000000000 | 2.882001701134740 | 25091.487117442600 | 575320.95618184700 |
| 53 | 1.5400 | 2.630000000000000 | 4.598821613335890 | 34168.335992336300 | 481386.08554235000 |
| 54 | 1.3300 | 2.860000000000000 | 4.201903212625970 | 49608.639985769500 | 361934.01122495800 |
| 55 | 1.5400 | 6.340000000000000 | 3.394653238396630 | 42726.812761730000 | 855602.66742478700 |
| 56 | 4.6600 | 1.540000000000000 | 3.557919339531340 | 45629.376010556700 | 440884.24408096300 |
| 57 | 2.0000 | 2.210000000000000 | 2.639575292354940 | 35138.691212280100 | 247467.08957789200 |
| 58 | 1.8400 | 2.620000000000000 | 2.150812173082370 | 39580.926397434900 | 532036.07450116700 |
| 59 | 2.1100 | 1.890000000000000 | 3.757223078937420 | 58203.118232827200 | 954107.13606633400 |
| 60 | 3.4800 | 28.000000000000000 | 2.116185361004610 | 67235.062120966500 | 357637.62578239700 |
| 61 | 7.5600 | 5.270000000000000 | 5.833276448847850 | 37988.475353631100 | 778972.51209085000 |
| 62 | 0.3800 | 2.340000000000000 | 3.308451043984030 | 60559.920819866300 | 623804.13204656500 |
| 63 | 2.0200 | 4.809000000000000 | 1.900780095645130 | 54329.514469353800 | 372955.85864408000 |
| 64 | 5.0900 | 2.970000000000000 | 4.958000357848040 | 41711.999424350600 | 1010810.78197689000 |
| 65 | 0.9300 | 3.530000000000000 | 2.776395727160170 | 31335.190455856200 | 491935.77870417700 |
| 66 | 3.1000 | 1.960000000000000 | 3.354781407677080 | 47711.417482418200 | 304527.56568607600 |
| 67 | 2.9100 | 5.410000000000000 | 3.838247269591880 | 49720.936283344600 | 475208.52878394600 |

**Table 6:** Metabolite levels per participant

| **Participant** | **Tryptophan (ug/mL)** | **Kynurenine (ug/mL)** | **Kynurenine/Tryptophan ratio** | **Quinolinic acid (ug/mL)** | **Kynurenic acid (ug/mL)** |
| --- | --- | --- | --- | --- | --- |
| 1 | 0.152380198661236 | 0.010311227355462 | 0.067667764224306 | 0.000986436498151 | 0.000002543990000 |
| 2 | 0.225767519324294 | 0.011492178016520 | 0.050902707576868 | 0.001392604248624 | 0.000030629600000 |
| 3 | 0.175124134265781 | 0.008346185963104 | 0.047658685069851 | 0.000804486000000 | 0.000044281096400 |
| 4 | 0.281124609633216 | 0.009506354367291 | 0.033815447106154 | 0.001977712196626 | 0.000008931761344 |
| 5 | 0.169674474976733 | 0.012565486769281 | 0.074056435247576 | 0.001488801054019 | 0.000001522290000 |
| 6 | 0.104672196985549 | 0.005680437643289 | 0.054268829802753 | 0.001557786188361 | 0.000005857544518 |
| 7 | 0.236851564360208 | 0.007858112938839 | 0.033177374023539 | 0.000841911199878 | 0.000112426000000 |
| 8 | 0.132646793354050 | 0.015887492851040 | 0.119772913082293 | 0.000240538806928 | 0.000015420390692 |
| 9 | 0.179304220085470 | 0.009102951787652 | 0.050768195992893 | 0.000663885000000 | 0.000011831071233 |
| 10 | 0.088573819880009 | 0.009908134330415 | 0.111863012612951 | 0.004560729716755 | 0.000009397083146 |
| 11 | 0.116156237449986 | 0.003394623902293 | 0.029224637237013 | 0.000177123000000 | 0.000009021780000 |
| 12 | 0.063610716814128 | 0.008820204420162 | 0.138659094912176 | 0.002980387836052 | 0.000001912539566 |
| 13 | 0.046920511211651 | 0.004507561608432 | 0.096068041290066 | 0.000630914826499 | 0.000009521090000 |
| 14 | 0.099354447496369 | 0.008432789416129 | 0.084875812091221 | 0.003353559000000 | 0.000020810800000 |
| 15 | 0.148477737449825 | 0.006008292018208 | 0.040465945409749 | 0.000382137000000 | 0.000006529865975 |
| 16 | 0.126962709478225 | 0.010890976755537 | 0.085780910003378 | 0.002534405232321 | 0.000000000000000 |
| 17 | 0.161386087892009 | 0.009490416924053 | 0.058805669361063 | 0.001158012700785 | 0.000023875045552 |
| 18 | 0.230294925057061 | 0.010068725642784 | 0.043721005316503 | 0.001073393995533 | 0.000010730333311 |
| 19 | 0.095262583375272 | 0.013296023168983 | 0.139572355670903 | 0.002980387836052 | 0.000007333820000 |
| 20 | 0.124970969808601 | 0.005860114184269 | 0.046891803698439 | 0.000907360406092 | 0.000012973500000 |
| 21 | 0.208334312918773 | 0.013997754651236 | 0.067188906403014 | 0.001729446446119 | 0.000014946200000 |
| 22 | 0.101596111495230 | 0.011241870029358 | 0.110652562031230 | 0.001669154566427 | 0.000004425810000 |
| 23 | 0.197590858727620 | 0.009713301867708 | 0.049158660123531 | 0.001012058570199 | 0.000000664885000 |
| 24 | 0.179091544685759 | 0.009007494807503 | 0.050295477786553 | 0.001647668393783 | 0.000042194092828 |
| 25 | 0.197197041139016 | 0.005453146142988 | 0.027653285827667 | 0.000439422473321 | 0.000016139878951 |
| 26 | 0.252836699148865 | 0.014911947518896 | 0.058978572213190 | 0.002084331576426 | 0.000010394500000 |
| 27 | 0.134557869914674 | 0.005797735814187 | 0.043087303759067 | 0.000928852000000 | 0.000003037110000 |
| 28 | 0.092888629064297 | 0.006776156006930 | 0.072949251971834 | 0.001144636234605 | 0.000006877560000 |
| 29 | 0.098008142244151 | 0.009688838192880 | 0.098857482358393 | 0.001295191201264 | 0.000088908900000 |
| 30 | 0.143400289577623 | 0.008946163274476 | 0.062385949852861 | 0.001028651949272 | 0.000037073700000 |
| 31 | 0.182088470405723 | 0.010453541617279 | 0.057409135207661 | 0.001557786188361 | 0.000027758038729 |
| 32 | 0.222868034429196 | 0.012605088637776 | 0.056558531016165 | 0.000700200057160 | 0.000020716600000 |
| 33 | 0.264416320613910 | 0.008678408142912 | 0.032821000317840 | 0.000692932000000 | 0.000013729063179 |
| 34 | 0.084504971431654 | 0.006012746400594 | 0.071152575981364 | 0.000320872773946 | 0.000003899700000 |
| 35 | 0.039479786054180 | 0.004158376757505 | 0.105329262721891 | 0.001216574000000 | 0.000009644560000 |
| 36 | 0.185681023868686 | 0.007819741575080 | 0.042113843472825 | 0.002881356000000 | 0.000012167700110 |
| 37 | 0.121449936669371 | 0.005146760510438 | 0.042377630253105 | 0.000335218939871 | 0.000003893929365 |
| 38 | 0.086593957776437 | 0.010923465180161 | 0.126145812717810 | 0.001718098000000 | 0.000011043000000 |
| 39 | 0.203538900590176 | 0.009289656233486 | 0.045640691811490 | 0.003722807804441 | 0.000005937180000 |
| 40 | 0.154690362217458 | 0.004561014564693 | 0.029484801116961 | 0.000356102298479 | 0.000007487830000 |
| 41 | 0.267649678017338 | 0.010140522171896 | 0.037887294492611 | 0.001633858721991 | 0.000030372100000 |
| 42 | 0.183179665839350 | 0.006656102023314 | 0.036336467766847 | 0.000877193000000 | 0.000005035570000 |
| 43 | 0.241958088305609 | 0.006907625220438 | 0.028548850211250 | 0.000715413618640 | 0.000005603325319 |
| 44 | 0.000000000000000 | 0.007094816261442 | N/A | 0.000902497093185 | 0.000001471194022 |
| 45 | 0.181327006880563 | 0.006261034355427 | 0.034528967654281 | 0.001130689000000 | 0.000019613900000 |
| 46 | 0.224476194926110 | 0.010363395906122 | 0.046167015213049 | 0.000727518822435 | 0.000001320020000 |
| 47 | 0.210167614752125 | 0.008157643503311 | 0.038814940698318 | 0.000841727000000 | 0.000021240800000 |
| 48 | 0.050276387723686 | 0.008582146916411 | 0.170699354209330 | 0.001977712196626 | 0.000007287140000 |
